# Limited Microvascular Remodelling Occurs in the Aged Human Hippocampus in Obstructive Sleep Apnoea

**DOI:** 10.3390/ijms262412040

**Published:** 2025-12-14

**Authors:** Cuicui Xu, Jessica E. Owen, Thorarinn Gislason, Bryndis Benediktsdottir, Jiming Ye, Stephen R. Robinson

**Affiliations:** 1 School of Health and Biomedical Sciences, RMIT University, Bundoora, VIC 3083, Australia; cuicui.xu@wimr.org.au (C.X.); jessica.owen811@gmail.com (J.E.O.); jiming.ye@hotmail.com (J.Y.); 2 Department of Sleep, Landspitali—The National University Hospital of Iceland, Áland 6, 108 Reykjavik, Iceland; thorarig@landspitali.is (T.G.); brynben@hi.is (B.B.); 3 Medical Faculty, University of Iceland, 101 Reykjavic, Iceland; 4 Institute for Breathing and Sleep, Austin Health, Heidelberg, VIC 3084, Australia

**Keywords:** ageing, angiogenesis, Alzheimer’s disease, CA1, capillary, CPAP, intermittent hypoxia

## Abstract

In mice, intermittent hypoxia is associated with an increase in microvessels in the hippocampus, whereas in humans with obstructive sleep apnoea (OSA), microvessels are lost from the heart and retina. The present study investigated microvascular changes in the hippocampus of patients with OSA, and whether patient age or use of continuous positive airway pressure (CPAP) influence microvascularisation. Using autopsy samples from 31 people with confirmed OSA, microvessels were immunolabelled and quantitatively analysed. Compared to the Low OSA group, the High OSA group had larger mean microvessel diameters in the fimbria and CA4, and greater mean microvessel length in the fimbria, which are indicative of microvascular remodelling. An absence of angiogenesis was indicated by similar mean vessel counts in both OSA severity groups. Increased age was associated with microvascular remodelling in the fimbria only. Treatment with CPAP was not associated with changed patterns of microvascularisation. We conclude that: (i) no evidence was found for angiogenesis in the human hippocampus in OSA or ageing; (ii) increased OSA severity is associated with microvascular remodelling in the fimbria and CA4; (iii) microvascular remodelling does not appear to be influenced by CPAP use; (iv) limited adaptability of the microvasculature may underpin the vulnerability of the hippocampus to hypoxic injury, particularly in severe OSA.

## 1. Introduction

Obstructive sleep apnoea (OSA) is characterised by recurrent episodes of upper airway collapse during sleep [1]. The repeated cessations of airflow are accompanied by transient drops in blood oxygen saturation [2], which in turn lead to arousals from sleep and sleep fragmentation [3,4]. Severe OSA typically involves more than 30 apnoeas per hour of sleep, with each episode lasting for an average of 20 s [5]. Obesity and older age are two major risk factors for OSA [6,7], and consequently the incidence of OSA is increasing worldwide, with a third of the adult population of the United States now estimated to suffer from this disorder, although many of these cases are undiagnosed [8]. The standard treatment for OSA is continuous positive airway pressure (CPAP), delivered through a mask worn during sleep, which prevents collapse of the upper airway, thereby reducing the severity of desaturations and supporting an uninterrupted sleep [9].

In addition to causing daytime sleepiness and increasing the risk of metabolic disorders [6], OSA can damage the brain [10,11]. Normal brain function depends on the regular supply of highly oxygenated blood, and repeated episodes of transient hypoxia can impair brain function and lead to neuronal injury, if the desaturations are long and deep enough. The brain region most sensitive to hypoxia is the hippocampus [12], which subserves various domains of memory and attention [13]. Untreated OSA is characterised by impairments in executive function, attention, cognitive flexibility, autobiographic memory, verbal memory, visual episodic memory and semantic memory [10,14]. Regular CPAP use improves cognitive flexibility but has no significant benefit for other cognitive domains [14]. A neuropathological investigation of the hippocampus in OSA [11] revealed significant reductions in tissue thickness and demyelination in areas that subserve episodic, spatial and semantic memory. Regular CPAP use reduced the tissue loss but not the demyelination [11].

In animals, acute hypoxia is a powerful inducer of cerebrovascular changes [15] that can involve the remodelling of existing capillary walls to increase their diameter and improve blood flow [16], or angiogenesis, which involves the budding of new capillaries from existing microvessels to increase the extent of the microvascular network [17]. The extent of microvascular remodelling and angiogenesis can be quantified from increases in capillary diameter, length, number and area fraction.

By delivering more oxygen to neurons, microvascular remodelling and angiogenesis represent important adaptations to acute episodes of hypoxia. Animal research using intermittent hypoxia (IH), a model of OSA, has confirmed that microvascular remodelling occurs in the hippocampus of mice exposed to IH. For example, when neonatal mice were exposed to 4 weeks of IH, the capillary density in the hippocampus was increased 1.29-fold [18]. A study of 4–5-month old mice [19] reported that 2 weeks of exposure to very severe IH increased the aggregate capillary length in the dorsal hippocampus by 1.4-fold. Finally, 8-week old mice exposed to 2 weeks of IH experienced a 1.4-fold increase in microvascular length within the dentate gyrus, but no significant changes in the CA1 or CA3 [20].

In contrast to animal studies, the presence of microvascular remodelling in the human hippocampus remains a matter for speculation because it has not been possible to verify whether such changes occur. Indeed, in severe OSA, the hippocampus loses substantial amounts of tissue [11], so if vascular remodelling does occur, it may have limited effectiveness at preventing neuronal loss. Microvascular remodelling has been observed in other organs of patients with OSA, however, these changes tend to be deleterious to the tissue. In the heart, for instance, OSA causes the microvasculature to dysfunction and provide less blood flow to cardiac muscle [21]. Furthermore, the choroid vascular bed of the retina shows degenerative changes, with a systematic reduction in its thickness as the severity of OSA increases [22]. This loss of microvasculature can be reversed with regular CPAP use [23].

The present authors were granted access to a unique bank of autopsied brain tissue obtained from people with a clinically verified history of OSA and CPAP use prior to their death. This study examined tissue from this bank to investigate whether microvascular remodelling or angiogenesis occurs in the hippocampus in OSA. Quantitative immunohistochemistry was used to investigate the association between OSA severity and microvascular remodelling in the hippocampus. Specifically, the present study investigated whether patient age or CPAP use influence microvascular changes in the hippocampus of patients with OSA.

## 2. Results

### 2.1. Descriptive Statistics

Descriptive statistics for the 31 patients are provided in Table 1. The mean ODI for the 15 mild–moderate OSA patients in the Low OSA group was 10.3 events/h sleep, while there was a significantly increased mean ODI of 42.4 events/h sleep for the 16 moderate–severe OSA patients in the High OSA group. These groups were comparable in terms of gender, age, BMI, and the interval between diagnosis and death.

**Figure 1:**
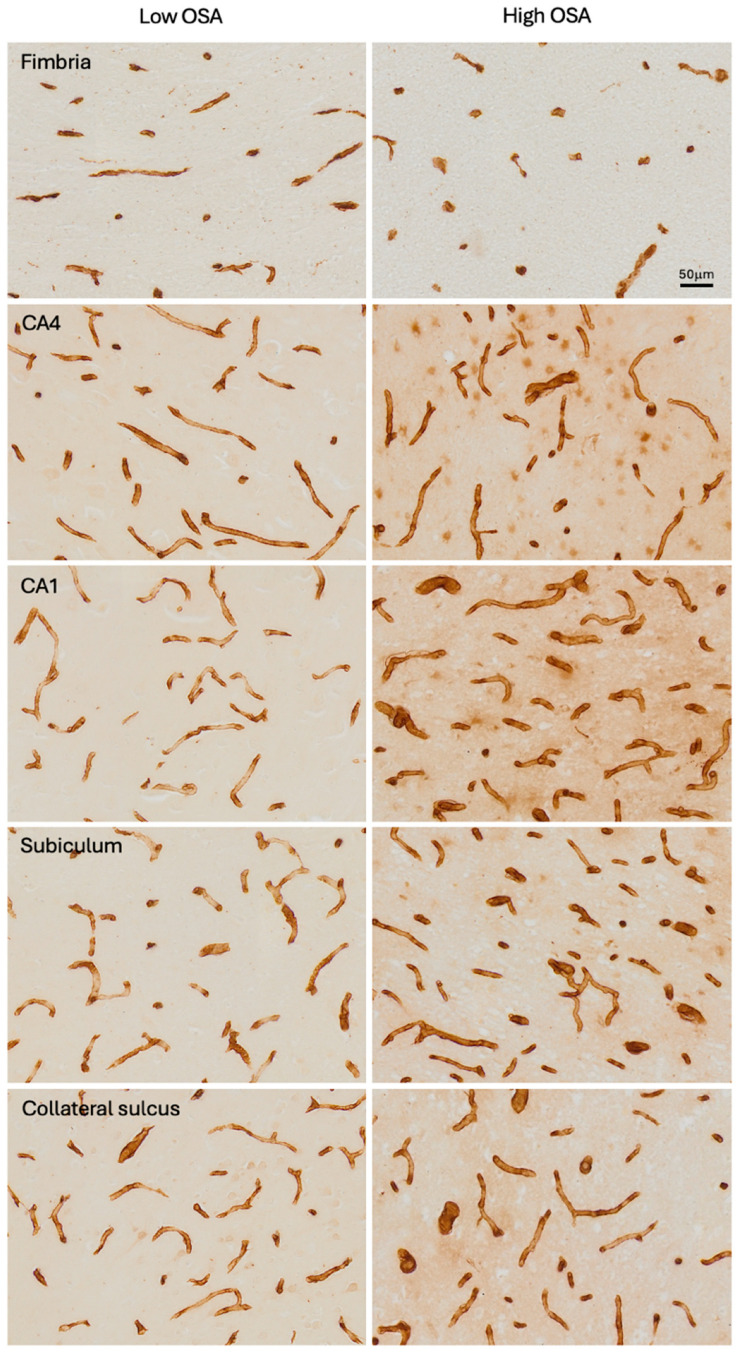
Photomicrographs of Collagen IV-immunostained microvessels in each of the five subregions analysed, in the Low OSA group (**left column**) and High OSA group (**right column**).

A significant correlation was found between OSA severity (ODI) and patient age (*r*^2^ = 0.175, *p* = 0.019). Figure 1 shows examples of immunohistochemistry staining of microvessels from the Low OSA (Figure 1, left column) and High OSA (Figure 1, right column) groups in each of the five regions examined. In some instances, brain tissue in the investigated regions was missing or damaged, and the final sample sizes were as follows: fimbria (n = 27), CA4 (n = 29), CA1 (n = 29), subiculum (n = 27), and collateral sulcus (n = 23).

### 2.2. Association Between OSA Severity and Microvascular Parameters

**Figure 2:**
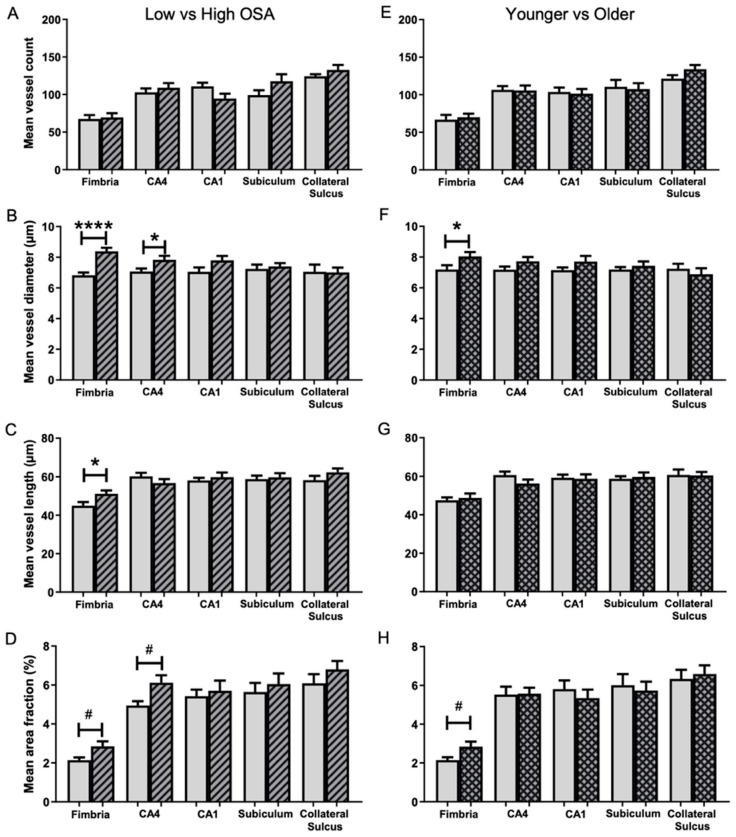
Comparison of microvessel parameters in five hippocampal subregions in the two OSA severity groups (**A**–**D**) and two age groups (**E**–**H**). Low OSA group (ODI < 20 events/h sleep; blank bars) and High OSA group (ODI ≥ 20 events/h sleep; striped bars). Mean microvessel count (**A**), mean microvessel diameter (**B**), mean microvessel length (**C**), and mean area fraction (**D**). Patients younger than 67.5 years (blank bars) and older than 67.5 years (grid bars). Mean microvessel count (**E**), mean microvessel diameter (**F**), mean microvessel length (**G**), and mean area fraction (**H**). Mean ± SEM. # *p* < 0.05 (unequal variance), * *p* < 0.05, **** *p* < 0.0001.

No significant differences were found in mean microvessel counts between the Low OSA group (ODI < 20 events/h sleep) and High OSA group (ODI ≥ 20 events/h sleep) for any of the five hippocampal regions examined (Figure 2A).

The mean microvessel diameter (µm) in the High OSA group was significantly larger than in the Low OSA group for both the fimbria (1.23-fold; 8.4 ± 0.2 vs. 6.8 ± 0.2, *t* (25) = 5.370, *p* < 0.001; Figure 2B) and the CA4 region (1.11-fold; 7.8 ± 0.3 vs. 7.1 ± 0.2, *t* (27) = 2.336, *p* = 0.027; Figure 2B).

The mean microvessel length (µm) in the fimbria was significantly greater in the High OSA group (1.14-fold) than in the Low OSA group (51.1 ± 1.8 vs. 45.0 ± 1.9, *t* (25) = 2.372, *p* = 0.026; Figure 2C). There were no differences between the two groups in the other four subregions.

The percentage of the full image occupied by labelled microvessels was investigated (Figure 2D). The High OSA group had a significantly greater area fraction than the Low OSA group in both the fimbria (1.33-fold; 2.85 ± 0.26 vs. 2.14 ± 0.14, *t* (20) = 2.401, *p* = 0.026) and the CA4 (1.24-fold; 6.12 ± 0.38 vs. 4.95 ± 0.22, *t* (22) = 2.649, *p* = 0.015). In the other three regions, there was a trend for area fraction to be higher in the High OSA group, but these differences did not reach significance.

### 2.3. Association of Age with Microvascular Parameters

The influence of age on angiogenesis and microvascular remodelling was investigated (Figure 2). The group of patients older than 67.5 years had larger mean vessel diameters in the fimbria (8.0 ± 0.3 vs. 7.2 ± 0.3, *t* (25) = 2.174, *p* = 0.039; Figure 2F) and a greater area fraction in the fimbria (2.84 ± 0.26 vs. 2.15 ± 0.15, *t* (21) = 2.342, *p* = 0.029; Figure 2H). There were no significant differences between the two groups with regard to mean microvessel count (Figure 2E) or mean microvessel length (Figure 2G) in any of the five hippocampal regions. ANCOVA’s were performed on the mean area fraction data for each of the five subregions, treating ODI as a fixed effect and age and BMI as covariates. These analyses revealed that the correlation between ODI and mean area fraction remained significant, even after correcting for patient age and BMI (*p* = 0.048). However, the correlation was no longer significant for the CA4 region (*p* = 0.079) and remained non-significant for the other three subregions.

ANCOVA’s were also performed on the mean area fraction data for CA4, treating ODI as a fixed effect and tissue thickness as a covariate. This analysis revealed that the correlation between ODI and mean area fraction remained significant, even after correcting for tissue shrinkage (*p* = 0.023). A similar analysis could not be performed for the fimbria, as tissue thickness data were not available for this region.

### 2.4. Association of Regular CPAP Use with Area Fraction

**Figure 3:**
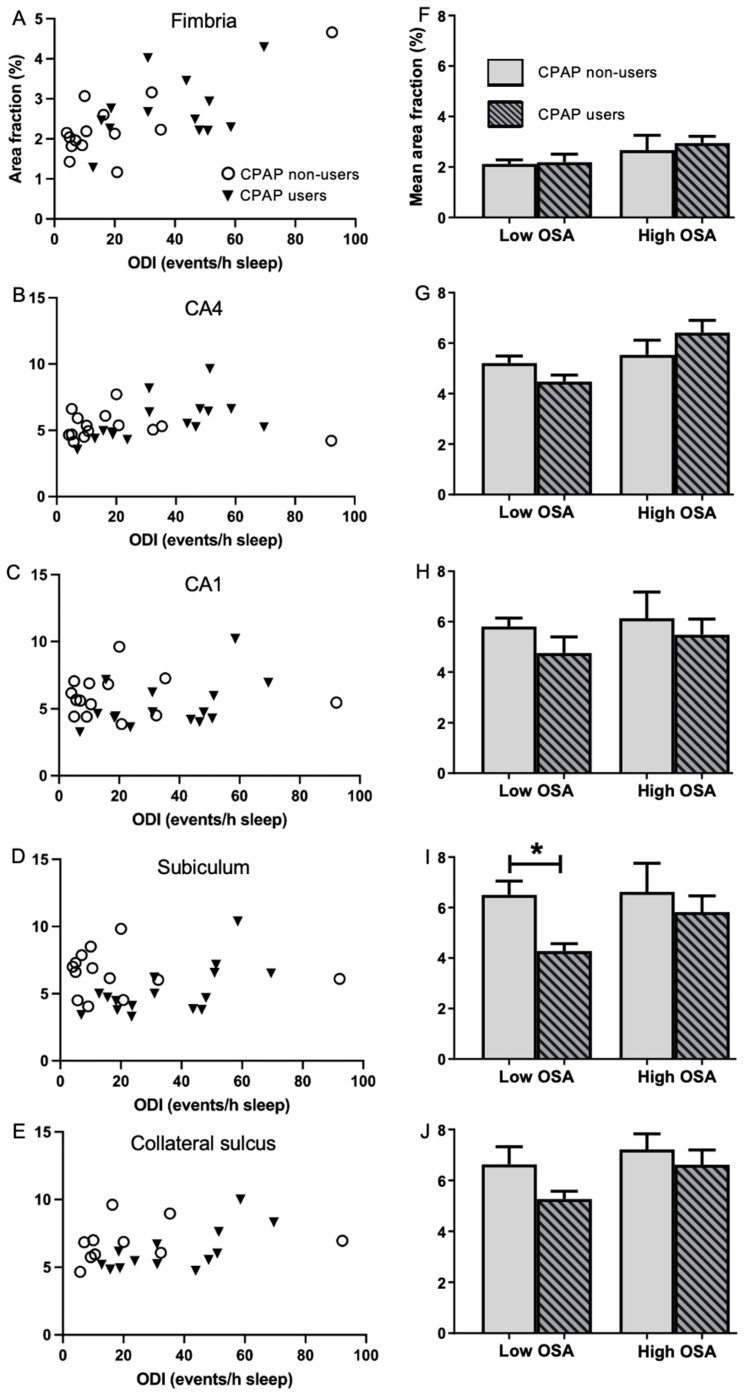
Comparison of area fraction in five hippocampal subregions in the CPAP non-user and user groups. **Left column:** Scatterplots of area fraction (%) in the CPAP non-users (open circles) and CPAP users (filled triangles) in the fimbria (**A**), CA4 (**B**), CA1 (**C**), subiculum (**D**) and collateral sulcus (**E**). **Right column:** Mean area fraction (%) in the CPAP non-users (blank bars) and CPAP users (striped bars), stratified into Low (ODI < 20 events/h sleep) and High (ODI ≥ 20 events/h sleep) OSA in the fimbria (**F**), CA4 (**G**), CA1 (**H**), subiculum (**I**) and collateral sulcus (**J**). Unpaired 2-tailed *t*-test between CPAP non-users vs. CPAP users. Mean ± SEM. * *p* < 0.05.

Changes in area fraction (%) demonstrate that there has been a reduction or increase in the amount of vascularisation of tissue. The association between regular CPAP treatment and the mean area fraction of microvessels was investigated by comparing CPAP non-users to regular CPAP users in the Low and High OSA groups (Figure 3). The mean area fraction in the subiculum was found to be significantly lower (4.28 ± 0.30 vs. 6.50 ± 0.55, *t* (11) = −3.003, *p* = 0.012; Figure 3I) in regular CPAP users than in CPAP non-users among the Low OSA group. There was a trend for area fraction to be lower in the CPAP user group, particularly in the CA1 and collateral sulcus (Figure 3C,E,H,J), but these differences did not reach significance. Among the High OSA group, no significant differences were found between regular CPAP users and CPAP non-users.

The primary findings of this study are summarized diagrammatically in Table 2.

## 3. Discussion

The present study used quantitative immunohistochemistry to examine four microvascular parameters in five distinct subregions of the hippocampus from 31 patients with a clinical history of OSA and CPAP use. Patients were divided into two groups, based on the median of OSA severity or the median of age. Statistical comparisons between those with less severe or more severe OSA revealed five significant microvascular differences. Comparisons between the younger and older groups revealed two significant differences, while comparison of CPAP users with non-users revealed one difference. These differences, and their implications, are discussed below.

Microvessels in the fimbria of the High OSA group had a significantly larger diameter and were longer than those in the Low OSA group, and these differences were reflected in a larger mean area fraction. Similarly, the CA4 of the High OSA group had larger microvessel diameters and a larger mean area fraction than the Low OSA group. These observations suggest that vascular remodelling occurred in the fimbria and CA4, but not in the CA1, subiculum or collateral sulcus. Notably, no subregion differed between groups with respect to mean vessel count, suggesting that angiogenesis did not occur in any of the five subregions. There was a non-significant trend towards an increased mean area fraction in the High OSA group for the CA1, subiculum and apex of the collateral sulcus; however, this trend was not observed for microvessel diameter or length, so this increase in area fraction may be an artifact caused by the thinning of cell layers in the High OSA group [11]. Collectively, these results imply that adaptive changes to the microvascular architecture are limited to a small portion of the hippocampus, involving the fornix and CA4. Although it is unclear why other subregions appear to be less adaptive, the likely consequence of this unresponsiveness is that these subregions will have greater vulnerability to episodes of intermittent hypoxia. As the present study lacked a healthy control group, we cannot exclude the possibility that microvascular changes occur in mild OSA and reach their maximum extent at very low ODI’s, although we saw no evidence to support this possibility (Figure 3). Conversely, it is possible that, despite the apparent lack of vascular remodelling, some microvessels in severe OSA may become diseased or lose their patency, in which case blood supply to the hippocampus could be compromised.

Compared to other brain regions, the hippocampus is particularly vulnerable to ischemia [24,25], with the CA1 region being most vulnerable [26,27]. Animal models of acute hypoxia have consistently reported the presence of neuronal apoptosis in the CA1 region [28,29,30]. The results of the present study suggest two reasons why the CA1 region is vulnerable. First, it had the lowest microvascular area fraction of the four grey matter subregions examined (5.5% vs. 6.0%, 6.0% and 6.5%), which means that the CA1 is likely to receive less blood than the other parts of the hippocampus. This observation accords with a magnetic resonance imaging study of 650 healthy adults that reported that CA1 had the slowest arterial transit time and lowest perfusion rate of the various hippocampal subfields [31]. Second, unlike the CA4 and fimbria, CA1 did not exhibit any increase in area fraction in the High OSA group. Indeed, CA1 showed no significant differences on any of the four vascular parameters for comparisons involving OSA severity, patient age or CPAP use. These observations suggest that the CA1 region not only receives less blood flow but is also more strongly constrained than other hippocampal subregions from undergoing microvascular remodelling in response to intermittent hypoxia.

When divided into two groups based on age at time of death, only the fimbria showed a significant difference. Mean vessel diameter was significantly larger in the older group, as was mean area fraction. These results imply that the fimbria undergoes microvascular remodelling with increasing age, and none of the subregions undergo angiogenesis. It seems unlikely that the age-related microvascular remodelling seen in the fimbria contributed to the vascular remodelling in the fimbria of the High OSA group, since both OSA groups were closely matched for age (Table 1). Furthermore, no age difference was observed for CA4, despite the evidence for vascular remodelling in this subregion in the High OSA group. Collectively, these results suggest that beyond the age of 50 or so (and possibly earlier), hippocampal microvessels may be incapable of remodelling in response to the diminished blood flow that characterises ageing [31]. This limitation may underpin the vulnerability of the hippocampus to the neurodegenerative diseases of ageing, such as Alzheimer’s disease.

Although the present study only found evidence for vascular remodelling (based on area fraction) in the fimbria and CA4, we need to consider the possibility that these outcomes could be due to the influence of other confounding variables such as patient age, BMI or tissue shrinkage. Indeed, ANCOVA analyses demonstrated that after controlling for patient age and BMI, the correlation between ODI and area fraction remained significant for the fimbria but not for CA4. This outcome suggests that the microvascular remodelling observed in CA4 might be attributable to factors other than OSA severity. On the other hand, ANCOVA analysis showed that tissue shrinkage does not appear to be a confounding factor in the correlation between ODI and area fraction in CA4. Although no data are available concerning the influence of tissue shrinkage in the fimbria, the area fraction results for the fimbria are mirrored by vessel diameter data. Since this variable is not influenced by tissue shrinkage, it seems more likely that the positive correlation between ODI and area fraction in the fimbria indicates genuine microvascular remodelling. The functional relevance of this remodelling is unclear at present. The fimbria is the main route followed by axons that leave the hippocampus, but if the neurons that contribute these axons are compromised by hypoxia (see below), the increased microvascularisation of the fimbria may provide limited benefit.

Hypoperfusion of the hippocampal microvasculature is one of the earliest regional changes detected during the course of Alzheimer’s disease [32], with the hippocampus having a perfusion rate that is almost half (57%) that seen in healthy controls [33]. A stereological analysis of microvessels in Alzheimer’s disease [34] found that mean capillary diameter in the hippocampus was 5% smaller than in healthy controls, indicating that these microvessels lack the capacity to increase their diameter in response to hypoperfusion. When considered together with the results of the present study, we speculate that the amnestic aspects of severe OSA and Alzheimer’s disease may share a common basis. Both conditions involve a loss of hippocampal volume [11], which seems to be due to an inability of the hippocampal microvasculature to undergo the remodelling needed to provide neurons with sufficient oxygen and nutrients.

CPAP use increases the thickness of the choroidal vascular layer in the retina [23]. To investigate whether regular CPAP use influences vascular remodelling in the hippocampus, data were first divided into Low OSA and High OSA groups then stratified by CPAP use/non-use. The non-CPAP group tended to have a higher mean area fraction in the Low and High OSA groups in the CA1, subiculum and collateral sulcus, although this difference only reached significance in the subiculum for the Low OSA group (which may have been a false positive). On the other hand, these comparisons were underpowered, so it is possible that more significant differences would have been detected if we had access to more autopsy cases. Taken at face value, the higher mean area fraction implies that regular CPAP use prevents vascular remodelling. However, the present study found no other evidence for vascular remodelling in the CA1, subiculum or collateral sulcus; furthermore, the fimbria showed the clearest evidence for microvascular remodelling, yet it was unaffected by CPAP use. These observations make it likely that the differences are due to another factor, such as tissue shrinkage, which can increase the area fraction occupied by microvessels, even in the absence of vascular remodelling [35]. Indeed our previous study, using tissue from the same autopsy series, demonstrated that regular CPAP use preserves the thickness of the cell layers in the hippocampus, whereas non-use of CPAP is associated with tissue shrinkage [11]. We conclude therefore, that unlike the choroidal capillary plexus of the retina [23], there is no consistent evidence for an influence of CPAP use on microvascular remodelling in the hippocampus. It remains possible, however, that if we had been able to stratify the data by hours of CPAP use per night, or by years of effective CPAP therapy, it might have been possible to discern an influence on microvascular modelling. It should also be noted that by grouping patients who never used CPAP with those for whom we had no user data, we introduced a potential misclassification bias that could have diluted any genuine CPAP effect. This problem could be overcome in the future by a much larger sample size.

Uncertainty exists regarding the relevance of mouse models of IH because many of the experimental parameters differ greatly from those seen in OSA [19,36,37]. These models typically use young mice, ranging from neonatal to four months of age; they use extreme levels of oxygen desaturation—sometimes for periods of 12 h/day—and the duration of these regimes is only 2–4 weeks. Furthermore, the haemoglobin oxygen desaturation profile in mice is very different to that in humans [19,37]. The findings from the present study may help to settle this uncertainty. Our investigation has shown that only the fimbria and CA4 have a significantly higher area fraction in the High OSA group (1.33-fold and 1.24-fold, respectively). The magnitude of these changes resembles the increases reported in mouse studies, which range from 1.29-fold to 1.4-fold [18,19,20]. Furthermore, Guan and colleagues [20] noted that only the dentate gyrus showed evidence of vascular remodelling, while Lim and colleagues [19] only observed vascular remodelling in extreme IH, and found no significant changes when the severity of desaturations more closely resembled those seen in severe OSA. In light of these similarities, we conclude that mouse models of IH can provide insights that align with neuropathological observations obtained from humans with OSA.

The findings of the present study, and those from animal studies of IH, indicate that the hippocampus has, at best, a limited capacity for vascular remodelling. This situation contrasts with other tissues such as the heart and retina, which can undergo extensive vascular remodelling in OSA [21,22], and it leads us to speculate on the reason for this difference. The encasement of the adult brain in a rigid skull provides very little potential for soft tissue expansion, as demonstrated by the lethal effects of cerebral oedema [38]. Perhaps the vulnerability of the adult brain to compression by an expanding microvascular network has led to the repression of molecular signalling pathways that promote microvascular remodelling and angiogenesis? In support of this novel concept, recent research in mice [39,40] has demonstrated that the pro-angiogenic genes that promote the formation of microvascular networks during brain development are epigenetically silenced in adulthood by histone deacetylase 2 (HDAC2) and the polycomb repressive complex 2 (PRC2). It remains to be demonstrated whether similar repression occurs in the human brain, and if it does, whether there are subregional differences within the hippocampus with respect to the extent of de-repression of these genes in OSA.

## 4. Materials and Methods

### 4.1. OSA Diagnosis

In Iceland, the diagnosis of OSA and CPAP treatment began in 1987. The OSA diagnoses are based on whole night polysomnography. OSA severity measurement has always been determined by the number of apnoeas and hypopnoeas per hour (AHI). More recently, the number of events involving a 4% decrease in oxygen saturation per hour (ODI) has been measured. Due to the archival nature of this study, not all AHI records were fully retrieved or comparable, whereas all of the ODI records were recovered. Therefore, ODI was used as the measure of OSA severity in the present study.

Exclusion criteria were as follows: clinical history of dementia, head trauma, stroke, multiple sclerosis, cerebral infection, pulmonary disease, as well as treatment for OSA other than CPAP. Blocks of hippocampal tissue containing the wall of the lateral ventricle, fimbria, parahippocampal gyrus and collateral sulcus were dissected by an experienced pathologist in Iceland. The blocks were post-fixed in 10% formalin, subsequently dehydrated in graded ethanols, embedded in paraffin wax in an identical orientation, and archived. Blocks that met the selection criteria for the present study were sent to RMIT University for analysis.

### 4.2. Study Samples

The hippocampal series examined in the present study consisted of autopsy tissue from 31 OSA patients (16 males and 15 females) who died between 1987 and 2014 [11], aged 42–89 years, with the average age at death being 67.5 years. To investigate the effect of patient age on microvascularisation, the study group was divided by median age. The ‘Younger’ group consisted of OSA patients aged <67.5 years (n = 16), while the ‘Older’ group consisted of those aged >67.5 years (n = 15).

The OSA severity (ODI) of these patients varied from very mild (4.1 events/h sleep) to very severe (92.2 events/h sleep). To aid analysis, the median ODI value of 20 desaturations/h sleep was used to divide the sample into two groups: ‘Low OSA’ group (ODI < 20 desaturations/h sleep; n = 15) and ‘High OSA’ group (≥20 desaturations/h sleep; n = 16).

Seventeen patients were confirmed to have used CPAP regularly until they died, although their nightly usage data were not available. Of the remainder, 3 patients were known never to have used CPAP, while 11 patients were not using CPAP at the time of death, although they may have used CPAP at some point between OSA diagnosis and death. To aid analysis, those patients who had regularly used CPAP were designated as the ‘CPAP user’ group (n = 17), while the remainder formed the ‘CPAP non-user’ group (n = 14).

### 4.3. Immunohistochemistry

Formalin-fixed paraffin-embedded tissue blocks were sectioned coronally at 20 µm to provide more complete visualisation of capillaries than the 5–10 µm thickness that is commonly used in animal studies. The immunohistochemistry protocol used by our laboratory has been described in detail elsewhere [11]. Briefly, sections were dried onto glass microscope slides, deparaffinised, rehydrated and then treated with antigen retrieval buffer. Slides were incubated with blocking solution before incubation with primary antibodies and then with secondary antibodies. A tertiary incubation with streptavidin-biotinylated horseradish peroxidase was followed by incubation in a solution of diaminobenzidine and hydrogen peroxide to visualise the chain of antibodies bound to the tissue. Immunolabelled sections were then washed, dehydrated and coverslipped with Depex. Control sections were processed in an identical way, except that the primary antibody was eliminated from the primary diluent. The diluents and incubation times are summarised in Table 3.

### 4.4. Histological Analysis of Hippocampal Microvasculature

**Figure 4:**
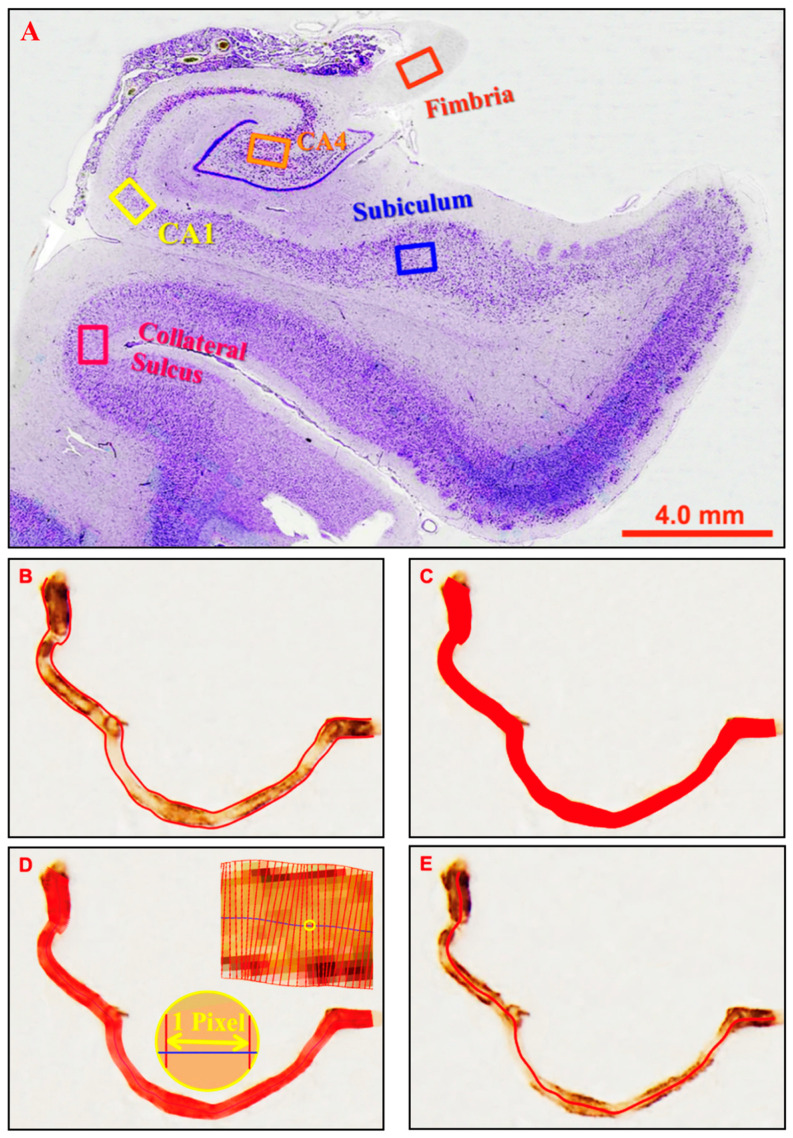
Micrographs illustrating the locations of the hippocampal subregions investigated and the parameters measured. (**A**) Micrographs were taken at five hippocampal locations as indicated by the frames in the cresyl violet-stained micrograph. (**B**) Vessel count was defined by the enclosed outline count of one vessel segment. (**C**) Area fraction (%) was defined as the proportion of the image area occupied by vessels. (**D**) Vessel diameter was defined as the mean diameter of a vessel segment when measured perpendicularly to the centre line at one-pixel intervals. (**E**) Vessel length was defined as the length along the centre line from the beginning to the end.

Five subregions of the hippocampus and overlying cortex were investigated: fimbria, Cornu Ammonis 4 (CA4 or hilus), Cornu Ammonis 1 (CA1), subiculum and collateral sulcus (near the apex), as illustrated in Figure 4A. These subregions were chosen because their location and appearance are distinctive, which enabled reliable identification, and they encompass the entire medio-lateral extent of the hippocampal formation and are therefore representative of this structure. The granule cell layer and molecular layer of the dentate gyrus were too narrow to permit reliable measurements of microvessels. Similarly, the CA2 and CA3 were excluded because: (i) their cross-sectional area was too small to permit reliable measurements; (ii) we could not reliably distinguish the boundaries of these subregions in immunolabelled sections; (iii) they were more commonly damaged during autopsy, so less complete series were available. Micrographs (‘images’) were taken at each of these five hippocampal subregions (unless a part of the tissue was missing) with an Olympus AX-60 microscope (Olympus Corporation, Tokyo, Japan) and Olympus DP73 camera (Olympus Corporation, Tokyo, Japan) at a final magnification of 400× (objective lens 10×, ocular lens 20×, magnification changer 2×) using Olympus CellSens software (CS-ST-V1.7; Olympus Corporation, Tokyo, Japan) at a resolution of 1600 × 1200 pixels. Subregions were identified by referring to adjacent cresyl violet-stained sections.

Four parameters were investigated: (1) Vessel count: the mean number of labelled profiles in an image; (2) Vessel diameter (µm): the mean diameter of all selected profiles in an image; (3) Vessel length (µm): the mean length of all selected profiles in an image; (4) Area fraction (%): the proportion of an image occupied by labelled profiles. Profiles were selected for analysis if their mean diameter was between 2–12 microns. This range encompasses capillaries but excludes most arterioles and venules. The lower limit ensured that diseased string vessels would be detected, although in practice, most labelled profiles are between 5–10 microns in diameter. For vessel count and area fraction, the original RGB (red-green-blue) micrographs were converted into greyscale images, auto-contrasted in Photoshop software and then analysed with Olympus CellSens software using the ‘Count and Measure’ feature. The threshold level was adjusted manually until all labelled profiles were selected, as determined by visual inspection. Profiles within the image, including those contacting a border, were selected if they were greater than 100 pixels in area. Vessel fragments that crossed an image border were included in their full extent. Data were then exported into Microsoft Excel for further analysis.

Due to a limitation of the algorithm in the CellSens software, which is designed for round/oval-shaped objects such as cells, the vessel diameter and length were analysed with software specifically developed for blood vessels: the Automated Retinal Image Analyzer (ARIA) v1.0 [41]. ARIA automatically recognises and generates a centreline and two edge lines along each vessel segment (Figure 4B). Vessel area was calculated from the number of pixels contained within the vessel outline (Figure 4C). Diameters for each vessel segment were measured perpendicularly along the centreline at 1 pixel intervals (Figure 4D). Vessel length was defined as the Euclidean distance along the centreline (Figure 4E). Detailed data were retrieved by a separate routine and then converted from pixels into micrometres (µm). Profiles selected by ARIA were visually inspected to ensure that that no labelled vessels within the nominated size range had been excluded and that no artifacts had been inadvertently included. If necessary, labelled profiles could be manually selected or deselected.

### 4.5. Statistical Analysis

Statistical analysis was performed using the IBM Statistical Package for the Social Sciences (SPSS version 21). Unpaired 2-tailed *t*-tests compared the Low OSA group to the High OSA group, CPAP non-users to regular CPAP users (n = 17), and OSA patients aged <67.5 years to those aged >67.5 years. *p* < 0.05 was considered significant (* with equal variances; # with unequal variances). GraphPad Prism 7 was used to generate graphs, presented as Mean ± SEM. In this exploratory study, we were interested in determining what differences might exist between groups. We chose not to adjust the *p* value to correct for multiple comparisons because we considered false positives (Type 1 errors) to be more benign than false negatives (Type 2 errors), which can occur if Bonferroni corrections are employed. Type 1 errors occur randomly (by definition), but the significant differences obtained in the present study are systematic, making it unlikely that these differences are false positives. Nonetheless, if any of these differences are false positives, it will further strengthen our contention that microvascular remodelling does not occur to any great extent in OSA.

## 5. Conclusions

The present research is limited by the retrospective nature of the autopsy series used. Specifically: (i) a modest sample size (31); (ii) a sample consisting entirely of Icelandic participants; (iii) the absence of a healthy age-matched control group; (iv) incomplete clinical records; (v) the autopsy samples are limited to the hippocampus and adjacent cortex. Nonetheless, strikingly similar patterns were obtained in the five hippocampal subregions, supporting the reliability of the methodology used, and providing confidence in the validity of our four main conclusions: (i) this study found no evidence for angiogenesis in the human hippocampus in OSA or ageing; (ii) increased OSA severity is associated with microvascular remodelling in the fimbria and CA4; (iii) microvascular remodelling does not appear to be influenced by CPAP use; (iv) the limited adaptability of the microvasculature may underpin the vulnerability of the hippocampus to tissue loss in severe OSA and in Alzheimer’s disease. The reasons for this limited adaptability are unclear at present and perhaps will be uncovered by investigations that employ markers of pericytes, basement membrane integrity, hypoxia-induced signalling or neuroinflammation. There is a need to investigate the repression of pro-angiogenic genes in the adult hippocampus, and whether there are subregional differences in the de-repression of these genes in OSA.

## Figures and Tables

**Table 1:** Descriptive statistics of the sample when divided by median OSA severity (ODI = 20 events/h sleep).

	Low OSA	High OSA	*p* Value
Number of patients	15	16	
Gender	M = 7, F = 8	M = 8, F = 8	
CPAP users	Y = 6, N = 9	Y = 11, N = 5	
Mean age at death (years)	66.0 ± 2.8	68.2 ± 1.7	0.589
Mean time lived after OSA diagnosis (years)	8.6 ± 1.2	6.8 ± 1.7	0.397
Mean BMI (kg/m^2^)	27.8 ± 1.0 (*n* = 14)	30.6 ± 2.1 (*n* = 13)	0.410
Mean ODI (events/h sleep)	10.3 ± 1.3	42.4 ± 4.9	0.001

Values represent mean ± SEM. M = male; F = female; Y = regular CPAP use; N = no/not sure for CPAP use.

**Table 2:** Summary of factors associated with microvascular remodelling in five subregions of the hippocampus.

Parameter	Region	OSA Severity	CPAP Use	Age
Vessel count	−	−	−	−
Vessel diameter	Fimbria	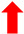	−	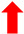
	CA4	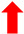	−	−
Vessel length	Fimbria	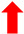	−	−
Area fraction (%)	Fimbria	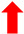	−	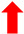
	CA4	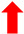	−	−
	Subiculum	−	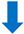	−

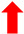
 = Increased; 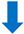 = Decreased; − = No change.

**Table 3:** Immunohistochemistry dilution protocol.

	Blocking (3 h)	Primary (16.5 h)	Secondary (4 h)	Tertiary (3 h)
1% BSA	Yes	Yes	Yes	Yes
4% Goat serum	Yes	Yes	Yes	
10% Triton X-100	Yes	Yes		
1% Ethanolamine	Yes			
Rabbit anti-collagen IV (Abcam Ab6586; Abcam Limited, London, UK)		1:20,000		
Goat anti-rabbit IgG (Vector BA-1000; Vector Laboratories, Newark, CA, USA)			1:300	
SB-HRP (GE Healthcare RPN 1051; GE Healthcare UK Ltd., Little Chalfont, UK)				1:300

BSA: Bovine serum albumin; SB-HRP: Streptavidin-biotinylated horseradish peroxidase.

## Data Availability

The original contributions presented in this study are included in the article. Further inquiries can be directed to the corresponding author(s).

## Abbreviations

AHI: Apnoea-hypopnoea index; CA1: Cornu Ammonis 1; CA4: Cornu Ammonis 4; CPAP: Continuous positive airway pressure; IH: Intermittent hypoxia; ODI: Oxygen desaturation index; OSA: Obstructive sleep apnoea; SEM: Standard error of the mean.
